# Synergistic Response Mechanisms in Rice Seedlings Exposed to Brown Planthopper Infestation and High-Temperature Stress

**DOI:** 10.3390/plants14111644

**Published:** 2025-05-28

**Authors:** Danyun Cao, Yuchen Ping, Yiru Lin, Jinyan Hu, Zimeng Wang, Wei Yuan, Tongtong Li, Linxin Liu, Bo Zhang, Shijiao Xiong, Cong Dang, Dawei Xue

**Affiliations:** 1 College of Life and Environmental Sciences, Hangzhou Normal University, Hangzhou 311121, China; cdddyun@163.com (D.C.); pingyc01@gmail.com (Y.P.); 13958806614@163.com (Y.L.); h574023292@outlook.com (J.H.); 15256196768@163.com (Z.W.); 13714031710@163.com (W.Y.); 2023111010042@stu.hznu.edu.cn (T.L.); lancerllx@163.com (L.L.); 2 National Key Laboratory of Rice Biology and Breeding, Institute of Insect Sciences, Zhejiang University, Hangzhou 310058, China; zhangbo2021@zju.edu.cn; 3 Xianghu Laboratory, Hangzhou 311231, China; xiongshijiao@zju.edu.cn; 4 Zhejiang Provincial Key Laboratory for Genetic Improvement and Quality Control of Medicinal Plants, Hangzhou Normal University, Hangzhou 311121, China

**Keywords:** synergistic response, transcriptome, brown planthopper, high temperature, stress, rice

## Abstract

Recently, rice yield has been severely affected by both brown planthopper (BPH, *Nilaparvata lugens*) infestation and high-temperature stress. Numerous previous studies have identified genes conferring resistance to BPH and high-temperature tolerance in rice, respectively. However, it remains unclear how rice synergistically responds to these two stress factors. In the present study, we found that pre-treatment with high temperature can enhance rice seeding resistance to BPH, while BPH feeding did not alter the high-temperature tolerance of rice. This result can be elucidated by the subsequent transcriptome analysis. Differentially expressed genes (DEGs) following high-temperature treatment were enriched in metabolic processes and phenylpropanoid biosynthesis pathways, thereby enhancing rice resistance to BPH. Further weighted gene co-expression network analysis (WGCNA) indicated that genes in the magenta and black modules were predominantly associated with the protein folding and transmembrane transport biological processes. And several candidate genes, including *Loc_Os01g02170* and *Loc_Os01g59870*, were identified that may play crucial roles in simultaneously regulating rice resistance to BPH and high-temperature stress. This research will provide new gene resources for cultivating rice with compound traits and provide ideas for the mechanism analysis of rice response to multiple stresses.

## 1. Introduction

Rice (*Oryza sativa*) is one of the most important food crops in the world, which feeds at least half the world population [1]. In natural paddies, rice will face a plethora of biotic and abiotic stresses at the same time, including biotic stresses such as pathogens, insects, pests and weeds, and abiotic stresses such as salt, drought, heavy metals, heat and cold, which seriously affect plant growth and crop yield, leading to large-scale yield reduction [2,3,4,5]. In order to cope with various stresses, plants have evolved a series of complicated molecular and physiological mechanisms [6]. Therefore, it is very significant for agricultural practice to understand how rice responds to multiple kinds of stresses and cultivate stress-resistant varieties.

Brown planthopper (BPH, *Nilaparvata lugens*) is a disastrous pest in rice cultivation. In recent decades, more than 70 BPH-resistance/QTLs genes have been detected in rice [7,8]. These BPH-resistant genes may enhance rice resistance to BPH by either influencing plant defense pathways such as salicylic acid (SA) and jasmonic acid (JA) or altering the volatile components and physical morphological structure of rice, thereby inducing certain levels of antibiosis or antixenosis against BPH [9]. For example, *OsBPH1/9* activates salicylic acid and jasmonic acid signaling pathways in rice and confers both antixenosis and antibiosis to brown planthopper [10]. *OsBph14* confers antibiosis resistance by inhibiting BPH feeding and reducing BPH growth rate and longevity [11,12]. Besides the above genes, a number of other pathway genes which were identified by the reverse genetics approach also participated in the pathway of rice resistance to BPH. *OsAOC* and *OsMYC2*, which are involved in the JA pathway, are significantly upregulated under BPH attack. BPH adult females laid more eggs on *osaoc* mutants than on WT plants, and the *osmyc2* mutants are susceptible to planthoppers in the field [13]. *OsEBF2* may reduce the content of ethylene in rice by inhibiting the expression of ethylene response factor genes and then positively regulates the rice resistance to BPH [14]. However, few of these BPH-resistant genes are associated with high-temperature stress in rice.

To mitigate the adverse effects of high-temperature stress, plants employ a diverse array of regulatory mechanisms. Heat shock factors (HSFs) and heat shock proteins (HSPs) play dominant roles in high-temperature stress. HSPs are regulated by HSFs and are induced in response to high-temperature stress. Some heat-responsive signal molecules (e.g., Ca^2+^, NO, hormones) also act upstream of high-temperature stress [15,16]. Cyclic nucleotide-gated ion channels (CNGCs) genes *OsCNGC14* and *OsCNGC16* affect Ca^2+^ influx to the cytosol in response to heat treatments [17]. Rice *Theromotolerance 2* (*OsTT2*), encoding a G gamma subunit, confers thermotolerance in rice during both vegetative and reproductive growth. A natural allele with loss of *OsTT2* function was associated with greater retention of wax at high temperatures and increased thermotolerance [18].

In recent years, some multiple stress response genes have been found and studied in rice. Transcription factors always involve multiple stresses. The expression of *OsWRKY45* was induced by infestation of the BPH, cold stress and drought stress. *OsWRKY45* negatively regulated the resistance of BPH, while silencing *OsWRKY45* enhanced the H_2_O_2_ and ethylene levels induced by BPH and improved the resistance of rice to BPH [19]. The expression level of *OsWRKY76* could be activated by both rice blast and low-temperature stress. Overexpression of *OsWRKY76* inhibited the basic resistance to rice blast, while increasing the cold tolerance. After inoculation with rice blast, the overexpressed plants of *OsWRKY76* withered [20]. Expression of *OsNAC6* was induced by *Magnaporthe oryzae.* The tolerance of overexpressing *OsNAC6* to rice blast was slightly improved [21]. Overexpressed *OsNAC6* under the control of root-specific promoters or constitutive promoters showed drought tolerance, while *osnac6* mutants showed drought sensitivity. This gene enhanced drought tolerance by mediating the adaptation of root structure, including the increase in root number and root diameter [22]. Rice calcium-dependent protein kinase *OsCPK10* was upregulated in rice leaves after rice blast infection; meanwhile, the expression of this gene was also induced by drought stress. *OsCPK10* could reduce the level of lipid peroxidation, protect the integrity of the cell membrane and enhance the detoxification ability of rice plants to H_2_O_2_ by regulating the accumulation of catalase protein. This gene could mediate the resistance to rice blast by reducing the accumulation of H_2_O_2_ during the growth of necrotizing fungi. This also improved drought tolerance of rice under the constitutive accumulation of *OsCPK10* [23].

In the actual situation of rice fields, multiple stress factors exist at the same time. However, in most previous studies, there was only one single stress factor targeted. Recently, due to climate warming, rice varieties evolution, insecticide resistance and other reasons, high temperature and BPH infestation have become the major stress factors affecting rice yield, and it still remains unclear how rice responds to these two stress factors. In this study, the response of rice seedlings to high temperature and feeding by BPH was analyzed by transcriptome sequencing technology, which provided new gene resources for cultivating rice with compound traits and provided ideas for the mechanism analysis of rice response to multiple stresses.

## 2. Materials and Methods

### 2.1. Plants and BPH Population

The rice plants used in this study were variety ZH11 (Zhonghua No. 11), which is always used as wild type in BPH-resistant and heat-stress gene functions. All rice plants were cultivated under 26 ± 1 °C (day and night), 80–90% relative humidity and a light/dark period of 14 h/10 h. The rice seedlings were sown in hydroponics boxes (12.5 cm in depth, 8.5 in width and 11.5 cm in height).

The *Nilaparvata lugens* population was fed on susceptible rice variety TN1 (Taichung Native 1) in a climate-controlled plant incubator at 26 ± 1 °C, 14 h/10 h light/dark cycle and 80% relative humidity.

### 2.2. Performance Observation of Rice Seedings Under Heat and BPH Stress

Fourteen-day individual rice seeding plants were infested with 15 3rd instar BPH nymphs. Five treatments were conducted in this experiment. Treatment 1: rice seedings were cultured at 25 °C for 12 days (25 °C: 12 d). Treatment 2: rice seedings were cultured at 38 °C for 12 days (38 °C: 12 d). Treatment 3: rice seedings were infested by BPH nymphs at 25 °C for 12 days (25 °C, BPH: 12 d). Treatment 4: rice seedings were infested by BPH nymphs at 25 °C for 1 day and then cultured at 38 °C for 11 days (25 °C, BPH: 1 d + 38 °C: 11 d). Treatment 5: rice seedings were cultured at 38 °C for 1 day and then infested by BPH nymphs at 25 °C for 11 days (38 °C: 1 d + 25 °C, BPH: 11 d). During these treatments, the survival condition of rice plants was recorded. Each treatment contained 8 replicates.

### 2.3. Chlorophyll Extraction

After these treatments finished, total chlorophylls were extracted using 45% ethanol, 45% acetone and 10% H_2_O at 4 °C for 12 h. The contents of chlorophyll were calculated using Arnon’s formula with absorbance of the supernatant measured at 663 nm and 645 nm using an ultraviolet spectrophotometer (Shimadzu, Kyoto, Japan).

### 2.4. Sample Collection, RNA Isolation and Transcriptome Sequencing

The whole rice seeding plants were sampled immediately after 8 h of high-temperature and BPH treatment, and each treatment contained 3 replicates. Total RNA was isolated using the Eastep^®^ Super Total RNA Extraction Kit (Shanghai Promega Biological Products, Ltd., Shanghai, China). The mRNA with polyA structure in total RNA was enriched by Oligo (dT) magnetic beads, and the RNA was interrupted to a fragment with a length of about 300 bp by ion interruption. After the library was constructed, PCR amplification was used to enrich the library fragments, and then the library was selected according to the fragment size, and the library size was 450 bp. Finally, these libraries were sequenced by the second next-generation sequencing (NGS) based on the Illumina sequencing platform.

The raw data for each sample were counted separately, including sample name, Q30, percentage of fuzzy bases, Q20 (%) and Q30 (%). The 3′ end joint is removed by Cutadapt, and the removed part has at least 10 bp overlap (AGATCGGAAG) with the known joint, allowing 20% base mismatch; reads with average mass fraction lower than Q20 were removed. The fragments per kilobase of transcript per million mapped reads (FPKM) values were screened by difference multiple |log_2_FoldChange| > 1. For the downstream analysis, in the case of differentially expressed genes (DEGs) expression, a cutoff at *p*-value of 0.05 was used for the up- and downregulated genes.

For each DEG, Gene Ontology (GO) and Kyoto Encyclopedia of Genes and Genomes (KEGG) enrichment analyses were conducted. Additionally, the proportion of DEGs enriched in a specific pathway was compared to the proportion of DEGs across all pathways. A DEG was considered significantly enriched when the *p*-value from the test was less than 0.05.

### 2.5. Quantitative Real-Time RT–PCR (qRT–PCR) Analysis

Total RNA was isolated by Eastep^®^ Super Total RNA Extraction Kit (Shanghai Promega Biological Products, Ltd., Shanghai, China). The 1000 ng total RNA of each sample was reverse-transcribed by Hifair^®^ III 1st Strand cDNA Synthesis SuperMix for qPCR (gDNA digester plus) kit (Yeasen Biotechnology Co. Ltd., Shanghai, China). Three independent biological samples were collected and analyzed. RT-qPCR was performed on the BIOER Line Gene 9600 Plus using Hieff^®^ qPCR SYBR^®^ Green Master Mix (No Rox) (Yeasen Biotechnology Co. Ltd., Shanghai, China). Primers used for target gene detection by RT-qPCR are listed in the Supporting Information, Table S1. Rice *ubiquitin* gene was used as internal reference, and 2^−△△Ct^ method was used to calculate the relative transcript levels of related genes [24].

### 2.6. Weighted Gene Co-Expression Network Analysis

The weighted gene co-expression network analysis (WGCNA) was constructed using the R software package WGCNA v1.72 [25]. The parameters were set as follows: -network type = unsigned, -soft power = 9, -module identification method = dynamic tree cut, -minimum module size = 30, -the threshold to merge modules with high similarity = 0.5. Ultimately, the target genes were clustered into 10 characteristic modules. Principal component analysis was performed on the genes in each module, with the first principal component defined as the module membership (MM), which characterizes the overall expression profile of the genes within the module. By treating tissue types as phenotypic traits, the correlation and statistical significance (*p* value) of each module’s MM with the trait indicators were calculated to identify key modules significantly associated with specific traits. In module analysis, gene significance (GS) was defined as the absolute value of the correlation between gene expression and the target trait. The novel regulatory gene interaction network was visualized using Cytoscape v3.9.1.

### 2.7. Statistical Analysis

The significance of variation in all treatments was evaluated by one-way ANOVA followed by Tukey’s HSD test. Statistical analysis was performed in Prism v. 8.0 software.

## 3. Results

### 3.1. Performance of Rice Seedings That Suffered Both Heat and BPH Stress

**Figure 1:**
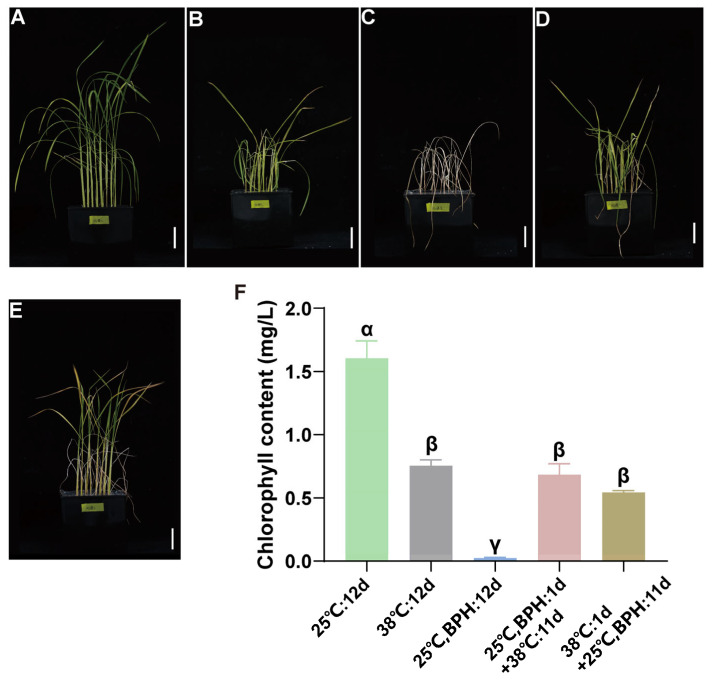
The performance of rice seedings that suffered both heat and BPH stress. (**A**–**E**) Different plant performances of rice seedings under five treatments; (**F**) chlorophyll content in rice seedings under five treatments. Mean ± SE, n = 8, different letters indicate significant differences between different treatments using Tukey’s HSD test (*p* < 0.05).

Rice seeding cultured at 25 °C was regarded as the control treatment (Figure 1A). During the seeding stage of rice plants under the dual stress of high temperature (38 °C) and infestation by brown planthoppers, it can be observed that after being infested by BPH nymphs for one day, and then suffering from high-temperature stress, the performance is similar with the treatment of heat stress without BPH infestation (Figure 1B,D,F). However, when rice plants are treated with high temperature for one day, and then exposed to BPH stress, they exhibited better growth when subjected to subsequent BPH infestation (Figure 1C,E), and the chlorophyll contents in these two treatments are consistent with the rice performances (Figure 1F). It is noteworthy that all BPHs died after being exposed to the sustained high-temperature condition for just 1 day; hence, the treatment in which rice seedings were exposed to both heat and BPH stress simultaneously for 12 days was excluded from this experiment. In summary, prior exposure to high temperature can enhance the BPH resistance of rice plants, while prior exposure to BPH could not change the heat resistance. The genes and mechanisms regulating these phenotypic adjustments need further in-depth investigation.

### 3.2. Transcriptome Analysis of Rice Seedings That Suffered Both Heat and BPH Stress

**Figure 2:**
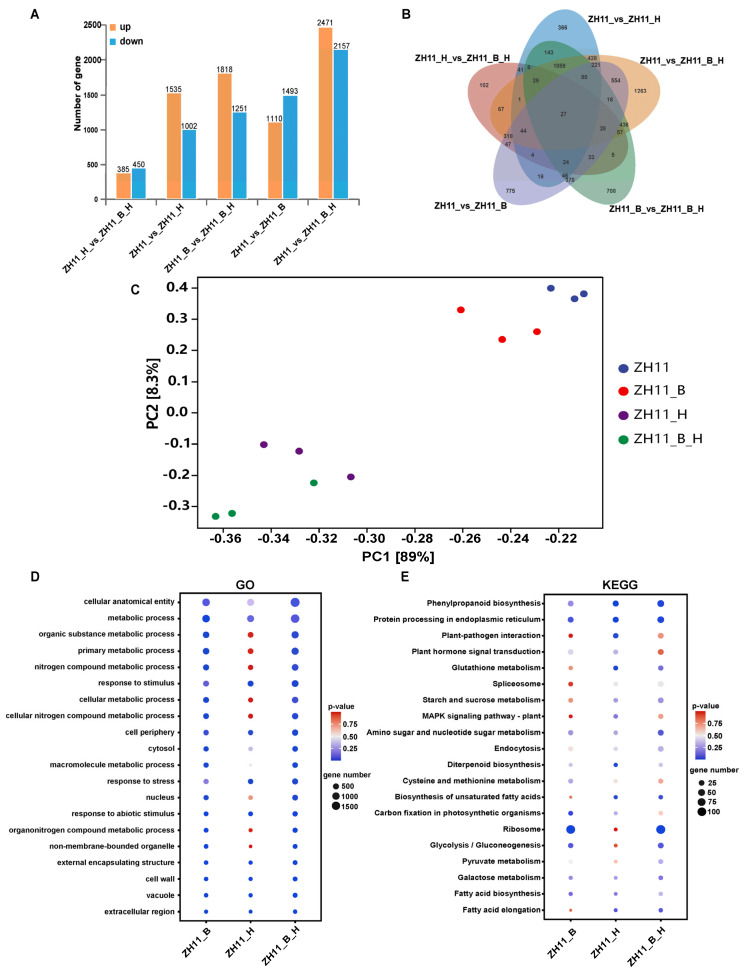
Transcriptome analysis of rice seedings that suffered both heat and BPH stress. (**A**) Histogram of differentially expressed genes (DEGs) from the transcriptome analysis; (**B**) Venn diagram of DEGs from the transcriptome analysis; (**C**) principal component analysis (PCA) of all the gene expression patterns; (**D**) top 20 terms of the DEGs GO enrichment analysis; (**E**) top 20 terms of the DEGs KEGG enrichment analysis.

To investigate the gene expression profiles of rice seedings that suffered both heat and BPH stress, we conducted transcriptomic analysis of plants subjected to four distinct experimental treatments: BPH-infested group (ZH11_B), high-temperature-stressed group (ZH11_H), both BPH-infested and high-temperature-stressed group (ZH11_B_H) and the untreated control group (ZH11). The results demonstrated that, compared to the control group, BPH infestation induced 1110 upregulated genes and 1493 downregulated genes, while high-temperature stress led to the upregulation of 1535 genes and downregulation of 1002 genes. When rice seeding suffered both BPH infestation and high-temperature stress, the number of upregulated and downregulated genes both exceeded 2000 (Figure 2A,B). The principal component analysis (PCA) revealed two distinct gene expression patterns corresponding to BPH infestation and high-temperature stress, respectively (Figure 2C), which indicated the transcriptomic data were solid.

Functional enrichment analysis of these DEGs revealed distinct biological patterns. GO enrichment analysis (Figure 2D) demonstrated that DEGs response to BPH infestation were primarily enriched in the pathways including cellular anatomical entity, metabolic process, macromolecule metabolic process, and so on. In contrast, DEGs induced by high-temperature stress were predominantly associated with metabolic process, response to stimulus, cell periphery and response to stress. When the two stress treatments were combined, DEGs showed enrichment patterns similar to BPH infestation, including cellular anatomical entity, metabolic process and macromolecule metabolic process. KEGG pathway analysis indicated that DEGs from both BPH-infestation and high-temperature-stress treatments were significantly enriched in protein processing in phenylpropanoid biosynthesis, ribosome and endoplasmic reticulum pathways (Figure 2E). To validate the accuracy of the transcriptomic data, some DEGs were selected to be verified by quantitative real-time PCR (qPCR) analysis (Figure S1), and most tested genes showed highly consistent expression patterns between qPCR results and RNA-seq data.

### 3.3. Analysis of the ROS Metabolic Regulation, SA and JA Pathways in Rice Seedings That Suffered Both Heat and BPH Stress

**Figure 3:**
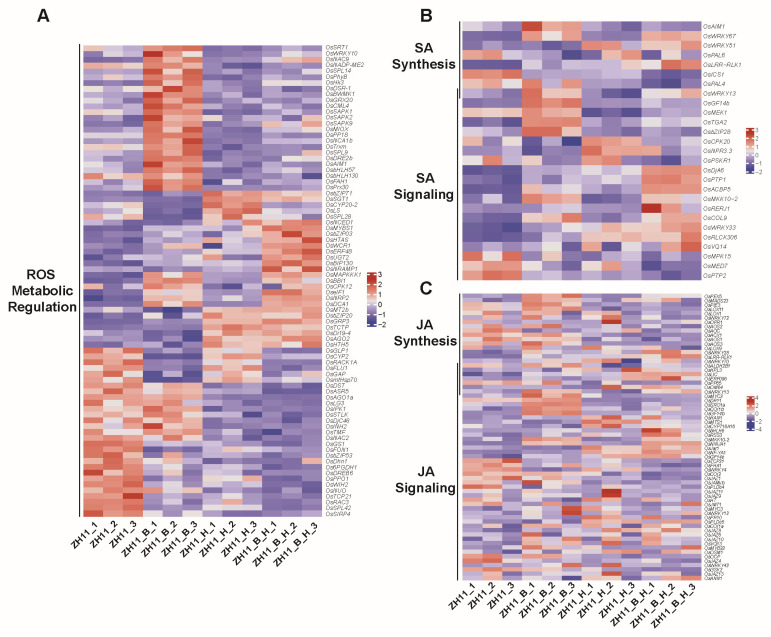
Gene expression in ROS metabolic regulation (**A**), salicylic acid (**B**) and jasmonic acid (**C**) pathways.

The enrichment analysis revealed a substantial number of DEGs in the reactive oxygen species (ROS), salicylic acid (SA) and jasmonic acid (JA) pathways under both stress conditions; thus, the DEGs in these three pathways were annotated and compared. In the ROS pathway (Figure 3A), genes such as the transcription factors *OsWRKY10*, *OsbHLH57* and stress-activated protein kinases *OsSAPK1*, *OsSAPK2* and *OsSAPK9* were significantly upregulated by BPH infestation, while their expression remained unaffected when they suffered high-temperature stress. In addition, high-temperature treatment induced significant upregulation of metallothionein *OsMT2b*, ARGONAUTE protein *OsAGO2*, transcription factors *OsbZIP20*, *OsbZIP71* and salicylic acid glucosyltransferase *OsSGT1*. These genes are also induced by the two-stress combination. Moreover, natural resistance-associated macrophage protein *OsNRAMP1* and ethylene-responsive transcription factor *OsERF48* are also significantly upregulated under both heat and BPH stress, whereas genes such as abscisic acid-stress-ripening-inducible 1 protein *OsASR5*, glutamine synthetase *OsGS1* and WINDHOSE protein *OsWIH2* are significantly downregulated by the two-stress combination.

In the SA pathway (Figure 3B), genes including DnaJ protein *OsDjA6*, protein tyrosine phosphatase *OsPTP1*, acyl-CoA-binding protein *OsACBP5*, WRKY transcription factor *OsWRKY67* and *OsWRKY33* are upregulated under combined BPH and high-temperature stress. Similarly, in the JA pathway (Figure 3C), genes such as WRKY transcription factor *OsWRKY28*, early responsive leucine-rich repeat receptor-like kinase *OsLRR-RLK1* and JA-amino acid synthetase *OsJar1* are upregulated under the combined stress conditions. In contrast, the expression levels of ROP GTPase *OsRAC3*, TCP family transcription factor *OsTCP21*, dehydration-responsive element-binding protein *OsDREB6* and protein tyrosine phosphatase *OsPTP2* are significantly downregulated under the combined stress treatment. These expression changes may be associated with the enhanced insect resistance observed in rice under high-temperature conditions.

### 3.4. Weighted Gene Co-Expression Network Analysis for the DEGs from Transcriptome

**Figure 4:**
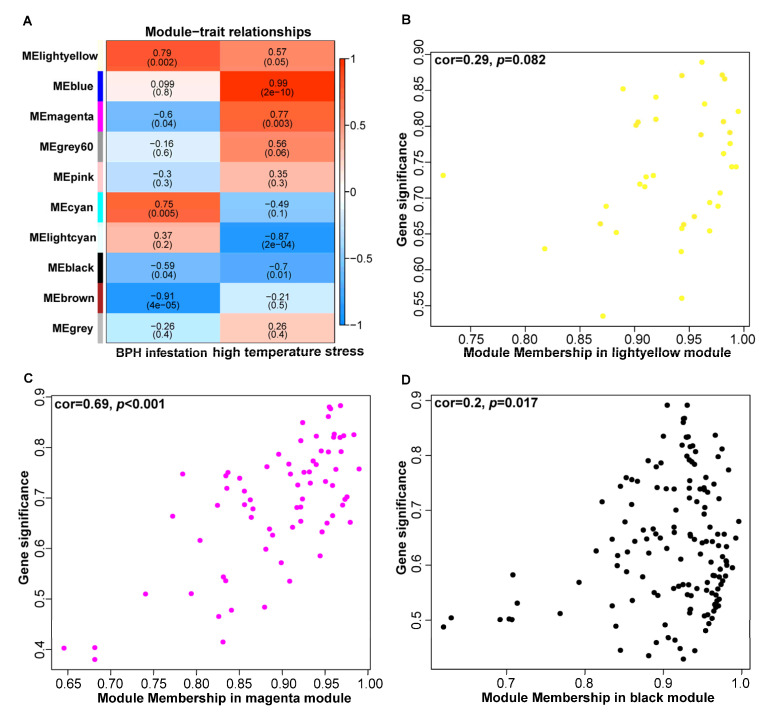
Weighted gene co-expression network analysis (WGCNA) for the DEGs. (**A**) Correlation heatmap between the modules and two phenotypic traits (BPH infestation and high-temperature stress); (**B**) correlation scatterplot between module membership and gene significance within the lightyellow module (cor = 0.29, *p* = 0.082); (**C**) correlation scatterplot between module membership and gene significance within the magenta module (cor = 0.69, *p* < 0.001); (**D**) correlation scatterplot between module membership and gene significance within the black module (cor = 0.2, *p* = 0.017).

To further identify the genes associated with resistance to BPH infestation and high-temperature stress, a WGCNA was performed based on all DEGs from transcriptome. According to the different gene expression patterns, 10 distinct color-coded modules were clustered. Among these modules, three modules, including lightyellow, magenta and black, showed significant correlations with both BPH infestation and high-temperature stress (Figure 4A). Within these color modules, both the magenta (cor = 0.69, *p* < 0.001) and black (cor = 0.2, *p* = 0.017) modules demonstrated significant correlations between module membership (MM) and gene significance (GS), while the lightyellow module exhibited a moderate correlation (cor = 0.29, *p* = 0.082) (Figure 4B–D). These results indicate that the magenta and black modules represent transcriptional signatures associated with rice responses to combined BPH infestation and high-temperature stress.

**Figure 5:**
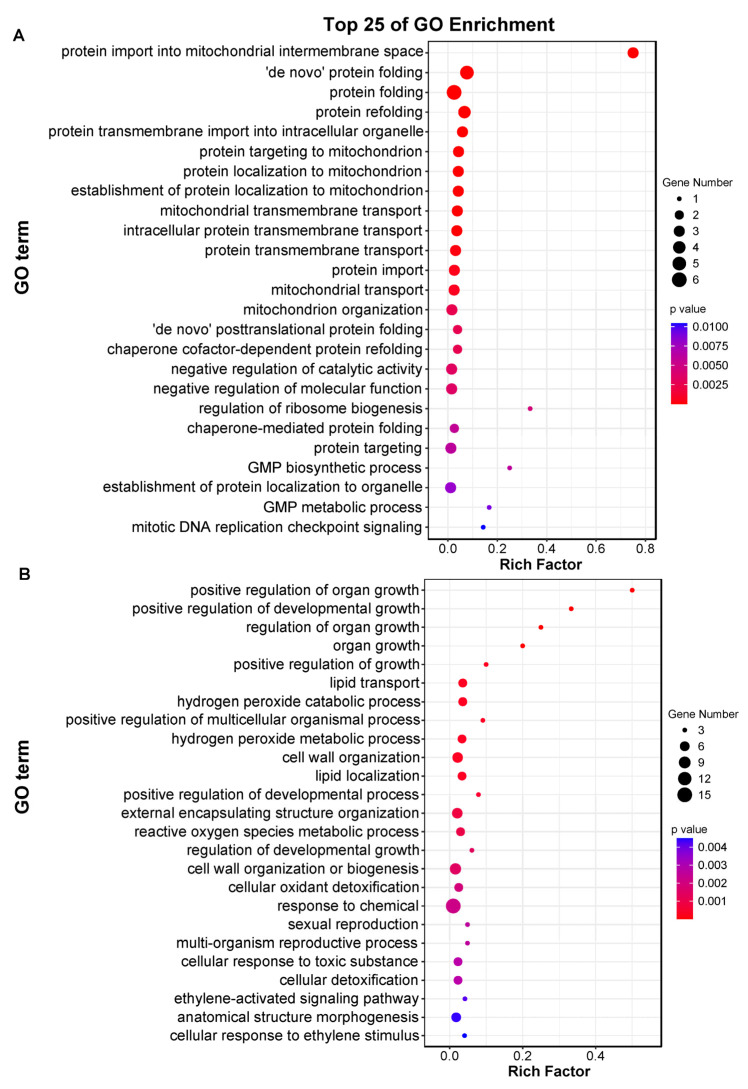
GO enrichment of the DEGs in the magenta (**A**) and black (**B**) modules.

**Figure 6:**
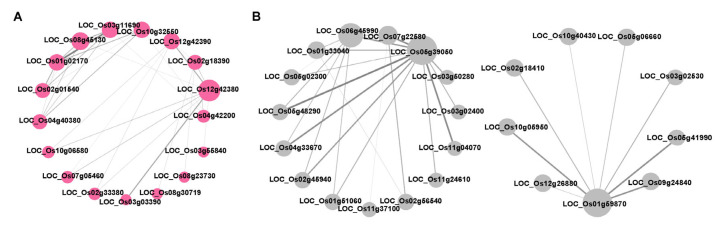
Gene functional network analysis in magenta (**A**) and black (**B**) modules.

Then, genes in the magenta and black modules were further analyzed. GO enrichment analysis demonstrated that genes within the magenta module were predominantly associated with the protein folding and transmembrane transport biological processes (Figure 5A). Meanwhile, the black module genes were significantly enriched in biological processes including cell wall organization, lipid transport and response to chemicals (Figure 5B). Network analysis of the 65 members in the magenta module was performed using Cytoscape (Figure 6A). By screening for high-weight members, we identified *Loc_Os01g02170*, *Loc_Os03g11690* and *Loc_Os12g42380* as core genes within this module. Similarly, in the black module, *Loc_Os01g59870*, *Loc_Os05g39050* and *Loc_Os06g45990* were identified as core genes (Figure 6B). These genes are likely to play crucial roles in rice responses to both BPH infestation and high-temperature stress, which need further functional characterization in future studies.

## 4. Discussion

Currently, in rice-growing regions, particularly in southern China, rice yield is severely affected by both high temperatures and brown planthopper (BPH). Numerous previous studies have identified genes conferring resistance to BPH and high-temperature tolerance in rice, respectively. However, there has been little research integrating these two types of stress factors, one biotic (BPH) and the other abiotic (high temperature). In the present study, we found that pre-treatment with high temperature can enhance rice resistance to BPH, but BPH feeding did not alter the high-temperature tolerance of rice (Figure 1). This result can be elucidated by the subsequent transcriptome sequencing data.

Differentially expressed genes (DEGs) following high-temperature treatment were enriched in metabolic processes and phenylpropanoid biosynthesis pathways (Figure 2D,E), which included metallothionein *OsMT2b*, ARGONAUTE protein *OsAGO2*, salicylic acid (SA) glucosyltransferase *OsSGT1*, transcription factors *OsbZIP20* and *OsbZIP71*, and other metabolism-related genes (Figure 3A). These genes have been reported to be involved in defense regulation in rice. For instance, *OsAGO2* can activate early defense immunity, such as the recombination of defense-related gene expression and the accumulation of reactive oxygen species, which may also be associated with BPH resistance [26]. *OsSGT1* encodes a salicylic acid glucosyltransferase, a key enzyme for SA metabolism, catalyzing the conversion of free SA to SA O-β-glucoside, and plays an important role in plant resistance regulation [27]. Phenylpropanoid is an important precursor for SA synthesis. High-temperature stress also led to a decrease in the transcription levels of SA synthesis pathway genes *OsPAL4* and *OsICS1* (Figure 3B), which are key genes for SA synthesis and may thus affect rice resistance to BPH. It has been reported that in *OsPAL4*-knockout rice, two flavonoid phytoalexins, sakuranetin and naringenin, are almost completely absent, and the levels of SA and jasmonic acid (JA) in the roots are also reduced, which may also lead to a decrease in rice resistance to BPH [28]. Meanwhile, calcium-dependent protein kinase *OsCPK20* was significantly induced by high temperature, and its high expression can also enhance rice’s immune capacity [29]. In the ribosome pathway, ribosome-inactivating protein *OsRIP1* was upregulated by BPH infestation, and the recombinant *OsRIP1* was toxic to brown planthoppers, suggesting that this ribosome pathway gene plays an important role in rice resistance to BPH [30]. Thus, high-temperature pre-treatment induced the expression of these BPH-resistance-related genes, thereby enhancing rice resistance to BPH. However, further validations of key metabolites and the accumulation of JA and SA are still required. 

Through transcriptome screening, we have also identified several genes that respond simultaneously to BPH feeding and high-temperature stress. In-depth exploration and functional analysis of these genes could lead to the development of new rice varieties that are resistant to both high temperatures and BPH. These genes include natural resistance-associated macrophage protein *OsNRAMP1* and ethylene-responsive transcription factor *OsERF48*, WRKY transcription factor *OsWRKY28*, early responsive leucine-rich repeat receptor-like kinase *OsLRR-RLK1* and JA-amino acid synthetase *OsJar1*, which have been reported to respond to various stresses. *OsNRAMP1* is involved in rice’s response to low-temperature stress [31]. *OsERF48* regulates the expression of calmodulin gene *OsCML16*, thereby enhancing rice’s drought tolerance. Overexpression of this gene can also affect physiological indicators such as superoxide dismutase, catalase and malondialdehyde, thereby enhancing redox homeostasis and membrane stability [32,33]. *OsWRKY28* positively regulates rice’s resistance to sheath blight, while negatively regulating resistance to blast and bacterial blight. In the mutant *oswrky28*, the contents of SA and JA are higher than in the wild type [34]. *OsLRR-RLK1* positively regulates resistance to the rice stem borer and may be involved in the perception of herbivore-associated molecular patterns [35]. *OsJAR1* is crucial for the accumulation of JA-IIe in crops when they are fed upon by pests. It catalyzes the synthesis of JA-Ile, the active form of JA that triggers the JA signaling pathway, also playing an important role in rice under high-temperature stress [36].

After the weighted gene co-expression network analysis (WGCNA), we identified several candidate genes that may play crucial roles in simultaneously regulating rice resistance to BPH and high-temperature stress. These genes include *Loc_Os01g02170*, *Loc_Os03g11690*, *Loc_Os12g42380*, *Loc_Os05g39050*, *Loc_Os06g45990* and *Loc_Os01g59870*. Notably, *Loc_Os01g59870* encodes lipid transfer protein *LTPL65*, which has been reported to be induced by BPH feeding [37]. Additionally, *LTP* family genes have been implicated in plant responses to high-temperature stress [38]. This suggests that *LTPL65* may have an important function in simultaneously responding to BPH feeding and high-temperature stress, warranting further in-depth investigation in the future.

However, when high-temperature and BPH stresses occur simultaneously, the entire process becomes highly complex. High temperature can affect not only plant growth but also the feeding behavior of herbivorous insects. It has been reported that high temperature can reprogram the JA signaling-mediated plant-induced defense. For instance, potato tuber moth *Phthorimaea operculella* grew heavier on leaves co-stressed by high temperature and insect herbivory than on leaves pre-stressed by herbivory alone [39]. And in warmer years, the damage caused by pests on temperature-sensitive plants is more severe [40]. However, in the present study, high-temperature pre-treatment enhanced rice resistance to BPH. Based on our analyses, high-temperature pre-treatment may have upregulated the expression of defense-related genes in rice. Therefore, when analyzing the interaction between rice responses to high-temperature and BPH-feeding stresses, the specific functions of each gene need to be investigated in depth. Moreover, there may be a certain balance between rice regulation of resistance to high temperature and BPH, in which phytohormones may play an important coordinating role in this balance mechanism.

Although we have identified some candidate genes that respond to both BPH infestation and high-temperature stress, their functional validation is still lacking. In the future, we will use gene editing techniques to create rice mutants of these genes, study their functions and regulatory networks, and conduct relevant phenotypic validation in the field. It should be noted that this experiment used sustained high temperature to identify some candidate genes. However, in the actual field situation, high temperature is not always sustained. In the future, we will also simulate the actual field environment, which includes periodic high temperatures, to further investigate the functions of these candidate genes. And the other rice growth stages, especially the reproductive growth period, will also be taken into consideration in the future.

## 5. Conclusions

In summary, under the escalating pressures of global climate change and intensive agricultural practices, rice cultivation is anticipated to confront increasingly complex biotic and abiotic stresses in field environments. To address these challenges, it is imperative to systematically investigate the functional roles of stress-responsive genes and their regulatory networks through advanced genomics, transcriptomics and metabolomics approaches. In this study, pre-treatment with high temperature can enhance rice seeding resistance to BPH, and it can be elucidated by the DEGs following high-temperature treatment, which were enriched in metabolic processes and phenylpropanoid biosynthesis pathways. Further WGCNA indicated that genes in the magenta and black modules were predominantly associated with the protein folding and transmembrane transport biological processes. And several candidate genes, including *Loc_Os01g02170* and *Loc_Os01g59870*, were identified that may play crucial roles in simultaneously regulating rice resistance to BPH and high-temperature stress. This research will enable the precise engineering of novel crop cultivars with multiplex traits, combining enhanced multiple stress resilience and yield potential.

## Data Availability

All data supporting this study are included in the article.

## Supplementary Materials

The following supporting information can be downloaded at https://www.mdpi.com/article/10.3390/plants14111644/s1, Figure S1: the verification of some DEGs by quantitative real-time PCR, Table S1: primers used in the quantitative real-time PCR experiment.
