# Supplementary material for: Synergistic Response Mechanisms in Rice Seedlings Exposed to Brown Planthopper Infestation and High-Temperature Stress
Source: Plants (Basel). 2025 May 28;14(11):1644. doi: 10.3390/plants14111644 (PMC12157819; doi:10.3390/plants14111644)
Supplement: Supplementary file 1 [file plants-14-01644-s001.zip › plants-3643299-supplementary.pdf]

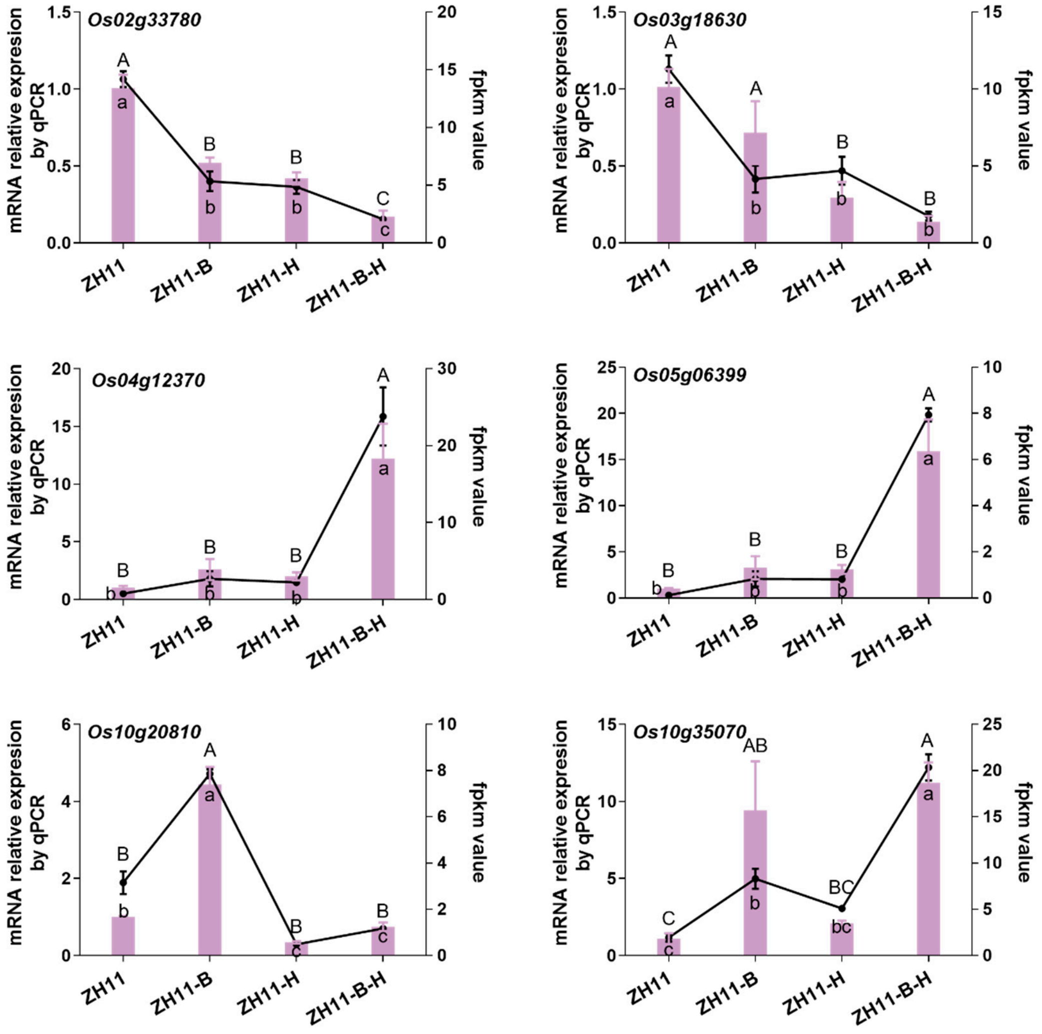

Figure S1 The verification of some DEGs by quantitative real-time PCR. One way ANOVA followed by Tukey HSD test was conducted. Different letters indicate significant differences between different treatments ($P<0.05$). Uppercase letters represent the comparative results of qPCR, while lowercase letters represent the comparative results of FPKM values from the transcriptome.

Table S1 Primers used in the quantitative real-time PCR experiment

Primer name	Forward Primer (5'-3')	Reversed Primer (5'-3')
q*OsUBQ*	CTGCTGCTGTTCTTGGGTTCA	TCATTATAGTTCTTCCATGCTGCTC
q*Os02g33780*	CAGAGAGCGAGGGATCAGAG	GTCTCAACCGCCATGACTTG
q*Os03g18630*	AGTTCAATGCCAGCTCGTTC	CTCCTGCTTGTCTTTCCTCC
q*Os04g12370*	GAGGGGAAATGGAGAGGAGG	ATCTTCTACAGCAGCGGGAA
q*Os05g06399*	ATCTTCTACAGCAGCGGGAA	GAGGGGAAATGGAGAGGAGG
q*Os10g20810*	ATATGCACCACACGAGAGCT	CGCCGGTGTTATGTCAGAAG
q*Os10g35070*	TGATGCACTTGTGTCAACCG	TGCTGTGAACATAATCGGCG
